# Axon regeneration after optic nerve injury in rats can be improved via *PirB* knockdown in the retina

**DOI:** 10.1186/s13578-021-00670-w

**Published:** 2021-08-11

**Authors:** Mei Yang, Lan Jian, Wei Fan, Xing Chen, Huan Zou, Yanming Huang, Xiaofan Chen, Yuan-Guo Zhou, Rongdi Yuan

**Affiliations:** 1 Department of Ophthalmology, Xinqiao Hospital, Army Medical University, 183 Xinqiao Zhengjie, Shapingba District, Chongqing, 400037 People’s Republic of China; 2 The Molecular Biology Center, State Key Laboratory of Trauma, Burn and Combined Injury, Research Institute of Surgery and Daping Hospital, Army Medical University, 10 Changjiang Zhilu, Chongqing, 400042 People’s Republic of China

**Keywords:** Regeneration, Müller cell, Optic, Retina ganglion cell injury, AAV

## Abstract

**Background:**

In the central nervous system (CNS), three types of myelin-associated inhibitors (MAIs) exert major inhibitory effects on nerve regeneration: Nogo-A, myelin-associated glycoprotein (MAG), and oligodendrocyte-myelin glycoprotein (OMgp). MAIs have two co-receptors, Nogo receptor (NgR) and paired immunoglobulin-like receptor B (PirB). Existing studies confirm that inhibiting NgR only exerted a modest disinhibitory effect in CNS. However, the inhibitory effects of PirB on nerve regeneration after binding to MAIs are controversial too. We aimed to further investigate the effect of PirB knockdown on the neuroprotection and axonal regeneration of retinal ganglion cells (RGCs) after optic nerve injury in rats.

**Methods:**

The differential expression of PirB in the retina was observed via immunofluorescence and western blotting after 1, 3, and 7 days of optic nerve injury (ONI). The retina was locally transfected with adeno-associated virus (AAV) *PirB* shRNA, then, the distribution of virus in tissues and cells was observed 21 days after AAV transfection to confirm the efficiency of *PirB* knockdown. Level of P-Stat3 and expressions of ciliary neurotrophic factor (CNTF) were detected via western blotting. RGCs were directly labeled with cholera toxin subunit B (CTB). The new axons of the optic nerve were specifically labeled with growth associated protein-43 (GAP43) via immunofluorescence. Flash visual evoked potential (FVEP) was used to detect the P1 and N1 latency, as well as N1-P1, P1-N2 amplitude to confirm visual function.

**Results:**

PirB expression in the retina was significantly increased after ONI. *PirB* knockdown was successful and significantly promoted P-Stat3 level and CNTF expression in the retina. *PirB* knockdown promoted the regeneration of optic nerve axons and improved the visual function indexes such as N1-P1 and P1-N2 amplitude.

**Conclusions:**

PirB is one of the key molecules that inhibit the regeneration of the optic nerve, and inhibition of PirB has an excellent effect on promoting nerve regeneration, which allows the use of PirB as a target molecule to promote functional recovery after ONI.

## Background

The optic nerve (ON) is a special central nerve formed up of the axons of the retinal ganglion cells (RGCs). Trauma, glaucoma, hypoxia and other factors can cause optic nerve injury (ONI) [1, 2]. The optic nerve is difficult to regenerate after injury, accompanied by the death of large numbers of RGCs in the retina that further causes visual dysfunction and even blindness [3, 4].

There are many reasons why optic nerve regeneration is difficult. In addition to the death of extensive numbers of RGCs after ONI, the lack of regeneration ability of RGCs, the inhibition effect of myelin-associated inhibitors (MAIs) on axon regeneration in the microenvironment, and colloidal scars are known to be key factors affecting axon regeneration [5–7]. Many studies have been carried out on the regeneration of an ON after injury using in vivo and in vitro methods. It was found that nerve growth factors [8], local resistance to glial scar [9], peripheral nerve transplantation [10], and other methods protect neurons or promote axon regeneration to a certain extent when used alone or in combination. However, it is still difficult to recover the full structural integrity and function of the ON. Therefore, the inhibitory effect of MAI on axonal regeneration has been the primary focus.

In the central nervous system (CNS), there are three MAIs, Nogo-A, myelin-associated glycoprotein (MAG), and oligodendrocyte-myelin glycoprotein (OMgp), that play an important role in inhibiting axonal regeneration [11]. They possess two co-receptors, Nogo receptor (NgR) and paired immunoglobulin-like receptor B (PirB) [12, 13]. Atwal [13] found that *NgR* knockout alone could not significantly reduce the inhibitory effect of Nogo-A on the neurite growth of cerebellar granule neurons in mice, while a single antagonist, PirB, could significantly reduce this inhibition. Kim [14] also found that inhibition of NgR did not cause a significant increase in CNS regeneration in NgR mutant mice. These suggest that NgR may not play a leading role in the inhibition of MAI on nerve regeneration.

PirB is derived from mice and has direct homology with human leukocyte immunoglobulin-like receptors (LILR) [15]. In the immune system, PirB is mainly expressed in B cells, macrophages, mast cells, and dendritic cells, and is a major histocompatibility complex class I (MHC I) molecule receptor. After binding to MHC I molecules, PirB led to the increased cytokine release by recruiting the tyrosine-protein phosphatase of the Src oncogene homologous domains 1 and 2 (SHP-1 and SHP-2) to the intracellular immunoreceptor tyrosine-based inhibition motif segment [16]. In the CNS, PirB is mainly expressed in the axons and synapses of neurons in the cerebral cortex, hippocampus, cerebellum, retina, and ON, which can combine with MAI to play an important role in inhibiting axon regeneration [17, 18]. In our previous study, we found that inhibition of PirB in primary cultured retinal Müller cells significantly promotes the regeneration of the co-cultured RGC axon [19]. However, it is unclear whether this effect is equally valid in vivo.

In this study, we aimed to further investigate the effect of PirB knockdown on the survival of retinal RGCs and axon regeneration in an animal model of ONI.

## Materials and methods

### Materials

#### Animals

Adult female Sprague–Dawley (SD) rats weighing 180-200 g were purchased from the Animal Center of Daping Hospital, Army Medical University, China. They were housed under standard laboratory conditions including a 12 h/12 h light–dark cycle and rodent chow and water.

#### Reagents

Reagents used in this study include cholera toxin subunit B (CTB) (Invitrogen, Poole, UK, Lot No: C34776), Nogo-A (R&D, MN, USA, Lot No: PK12319031), and Super ECL Plus (US Everbright®Inc., Suzhou, China, Cat No: S6009). Adeno-associated viral vector (AAV) PirB shRNA (with green fluorescent protein (GFP) tag) target sequence was as follows: GGAGCCGAACTTTATTGTCTCTATA (Hanbio Co. Ltd., Shanghai, China). The antibodies used are listed in Table 1.

**Table 1:** List of primary and secondary antibodies used for western blotting and immunohistochemistry (IHC)

Antibody	Source	Lot/Cat No.	Dilution	
Primary antibodies			WB	IHC
Goat anti-PirB	R&D, Minnesota, USA	AF2754	1:1000	1:100
Rabbit anti-Vimentin	Abcam, Cambridge, UK	ab92547		1:500
Rabbit anti-Phospho Stat3 Y705	Abcam, Cambridge, UK	ab76315	1:5000	
Rabbit anti-Stat3	Abcam, Cambridge, UK	Ab68153	1:2000	
Rabbit anti-GFP	Abcam, Cambridge, UK	Ab290		1:200
Rabbit anti-GAP43	Cell Signaling, Boston, USA	#8945		1:200
Rabbit anti-CNTF	Abcam, Cambridge, UK	ab175387	1:1000	
Rabbit anti-beta III Tubulin	Abcam, Cambridge, UK	ab18207		1:500
Mouse anti-beta III Tubulin	Abcam, Cambridge, UK	ab78078		1:500
Mouse anti-GAPDH	Abcam, Cambridge, UK	ab8245	1:5000	
Secondary antibodies				
HRP-labeled anti-mouse IgG	Invitrogen, Poole, UK	G-21040	1:10,000	
HRP-labeled anti-rabbit IgG	Invitrogen, Poole, UK	31,460	1:10,000	
HRP-labeled anti-Donkey IgG	R&D, Minnesota, USA	HAF109	1:1000	
Alexa488 anti-goat IgG	Invitrogen, Poole, UK	A32814		1:300
Alexa594 anti-rabbit IgG	Invitrogen, Poole, UK	A32740		1:300

### Methods

#### ONI model

Adult female SD rats were divided into three groups with four rats in each group as follows: Blank group (without treatment); sham group (sham operation); ONI group (optic nerve clamp injury). The ON clamp injury was established according to the previously reported method by our group [18]. Briefly, the rats were anesthetized via intraperitoneal injection of 2% pentobarbital sodium (50 mg/kg), followed by a drop of 4% oxybuprocane hydrochloride eye drop into the conjunctival sac for surface anesthesia. A 5 mm horizontal incision was made in the lateral canthus of the superior temporal quadrant 1.5 mm from the limbus of corneosclera. The subcutaneous tissue layers were separated bluntly, and the ON was fully exposed for 3–5 mm. At the 2 mm post bulbar, the ON was clamped with a small artery clamp perpendicular to the longitudinal axis of the ON for 15 s, and the clamping force was 112 G. Along with the clamp injury, the pupil of the injured eye gradually expanded. After the operation, the eyeball fascia was sutured layer by layer, and an appropriate amount of chlortetracycline eye ointment was applied to the conjunctival sac. At the same time, the contralateral eye was operated on in the sham operation control group, but the ON was not clamped. The success of modeling was judged via intraoperative pupil dilation, and the absence of postoperative hemorrhage, infection, cataract, and other complications.

#### Vitreous cavity injection [20]

Adult female SD rats were divided into four groups with four rats in each group as follows: PBS group (negative control); Nogo-A group (positive control); AAV shRNA group (empty viral control); AAV PirB shRNA group. AAV (1.0 × 10^9^ μg /μL, viral final titer), CTB (1 μg/μL), and Nogo-A (0.1 μg/μL) were prepared according to the manufacturer’s instructions. After anesthesia, one drop of 0.5% compound tropicamide eye drops was applied to each eye, and the pupil was dilated for 20 min. Exactly 2 μl of PBS, Nogo-A, AAV shRNA, and AAV PirB were collected using a microsyringe (Hamilton, Switzerland) with a diameter of 0.33 μm, then applied into the vitreous cavity from the superior temporal quadrant, 1.5 mm away from the corneoscleral edge, and the needle tip was toward the optic papilla to avoid puncturing the lens. It was obvious from the dilated pupil that the needle tip was located in the vitreous cavity and the drug was slowly injected (injection time was more than 1 min). After injection, the needle tip was left in the eye for about 20 s to adjust the volume of the eyeball. Then the needle was pulled out and the puncture port was immediately pressed with a cotton swab for about one minute to prevent drug leakage. The injection method for CTB was the same, but the injection time was two days before each animal was killed.

#### Whole-mount retina preparation

According to the previously published method [21], rats were anesthesized, 0.9% NaCl (4℃) was used to drain the blood from the heart, then, 4% polyformaldehyde was used to fix the tissue. The eyeball was quickly removed, soaked in 4% polyformaldehyde, and fixed on ice for 2 h. The eyeball was transferred to precooled PBS, and the retina was carefully separated. Intact retinas were dissected into four sections like a clover and then soaked in methanol (4 °C) for 1 h. Samples were washed with PBS 3 times for 10 min, neutral gum seal was observed under a laser confocal microscope (Leica, Weztlar, Germany). At 200 × magnification, the retina was examined for RGCs at 2/6, 1/2, and 5/6 of the radial distance from the optic papilla. RGC identification rate was calculated as follows: The number of RGCs in the experimental group/ that of RGCs in the control group. CTB-positive cells were identified as RGCs, which were counted in 12 regions (three images per retinal quadrant) per retina, and the number was averaged to estimate the overall RGC survival [22].

#### Frozen sections and immunofluorescence

After anesthesia, the rats were killed via cervical dislocation. The eyeball and ON were quickly removed and embedded in Tissue-Tek OCT compound, and then placed in the refrigerator at – 80 °C for 20 min. The tissue was cut into 10 μm sections using frozen section machine (Leica, Weztlar, Germany), fixed with 4% paraformaldehyde for 15 min, washed three times with PBS for 5 min, blocked in 0.1% Triton × 100 and 10% goat serum for 30 min at room temperature (20–22 °C), and primary antibodies were added before incubation at 4 °C overnight. Samples were then washed with PBS 3 times for 5 min, secondary antibody was added and incubated in the dark for 1 h at room temperature, followed by counterstaining with 4′,6-diamidino-2-phenylindole (DAPI) for 15 min. Axon growth was quantifified by counting the number of GAP43-positive axons extending 200, 500, 1000 and 1500 µm from the crush site in three sections per case (20 × magnification). Values were normalized referencing the formula described formerly [23], ∑*a*_d_, the total number of axons extending distance which was then averaged over three sections per case; *πr*^2^, the area of the cross-sectional width of a nerve; *t*, the thickness of Sections (10 μm):

$$\sum a_{{\text{d}}} = \uppi r^{{2}} \times [{\text{average axons}}/{\text{average nerve width}}]/t$$

#### Flash visual evoked potential (FVEP)

To evaluate the function of the optic nerve, FVEP was measured 28 days after ONI using the visual electrophysiology system (IRC Medical Equipment Factory, China). [24, 25] After anesthesia, rats’ pupils were dilated with 0.5% compound tropicamide eye drops. The electrode at the primary visual cortex was considered the active electrode, the electrode at the frontal cortex was considered the reference electrode, and the ground electrode was inserted into the tail. While one eye was being tested, the other was covered with a piece of opaque cloth. Three stable waveforms were recorded for each animal. Test parameters were as follows: the intensity of the optical stimulator was 3.0 cd^2^S/m^2^; the background light intensity was 30 cd^2^S/m^2^; the pass frequency was 1–100 Hz, the stimulation frequency was 2 Hz; each item was superimposed 45 times. Detection index was as follows: N1 and P1 latency, as well as N1-P1 amplitude (N1 bottom to the peak of P1); P1-N2 amplitude (P1 peak to the bottom of N2).

#### Western blotting

According to the previously described method [19], equal amounts of protein (40 µg) were separated via 10% SDS-PAGE and transferred onto polyvinylidene fluoride membranes. After blocking with 5% skimmed milk for 2 h, primary antibodies were added for incubation on a shaker at 4 °C overnight. Then, secondary antibodies were added at room temperature for 1 h. Enhanced chemiluminescence was used for development before detection on an Omega Lum G gel imager (Aplegen, Pleasanton, USA). Gray values were analyzed with the Image J software (National I1stitute of Health, Bethesda, Germany), and GAPDH or Stat3 were used as an internal reference. The ratio of the gray value of the target strip to that of the internal reference was calculated.

#### Statistical analysis

SPSS 19.0 (IBM, Armonk, USA) was used to analyze the data, all results were expressed as the mean ± standard deviation. A two-sided Student’s t-test was used for two-group comparisons, and multiple group comparisons were performed using ANOVA followed by a post hoc Student’s t-test in cases with homogeneity of variance and normal distribution. For all other cases, a nonparametric test was used, *p* < 0.05 was considered statistically significant.

## Results

### *PirB* expression increased in the retina after ON clamp injury

**Fig. 1:**
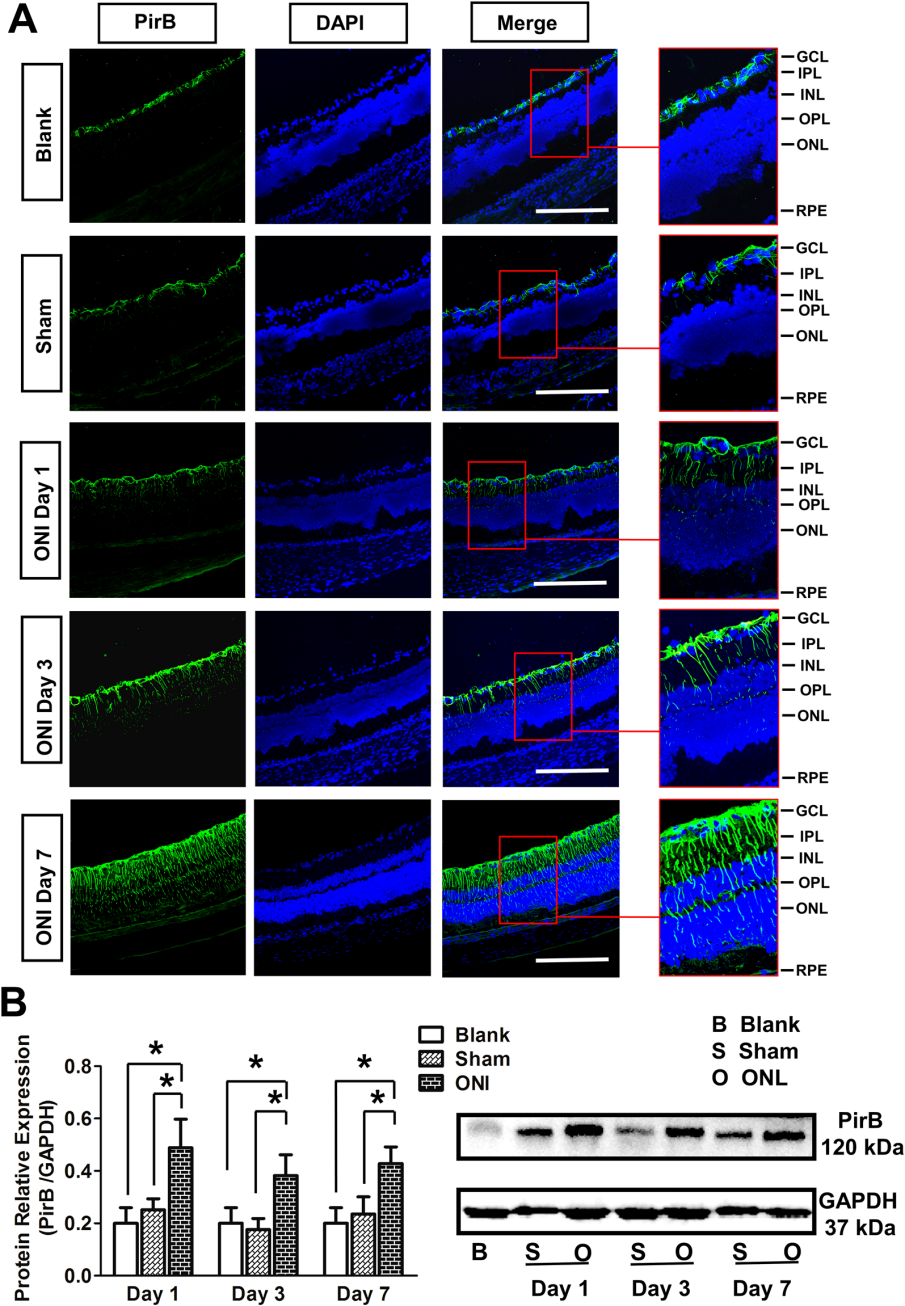
*PirB* expression in the retina after ONI. **A** PirB (green, 488) expression in the retina after ONI obtained via immunostaining. DAPI represents nuclear staining (blue). ONI, optic nerve injury; GCL, ganglion cell layer; IPL, inner plexiform layer; INL, inner nuclear layer; OPL, outer plexiform layer; ONL, outer nuclear layer; RPE, retinal pigment epithelial cell layer; scale bars: 250 μm, n = 4. **B** The target strip and bar charts showing PirB expression in the retina tissue. GAPDH was used as an internal reference. The ratios of gray values of target proteins to those of respective internal references are provided. The experiment was repeated three times (n = 4). Data are presented as the mean ± standard deviation. One-way ANOVA was performed, and Student’s *t*-test was used for group-pair comparisons. *, *p* < 0.05

The results of immunofluorescence showed that PirB (green, 488) was mainly expressed in the ganglion cell layer (GCL) of the retina in the blank group. It was mainly expressed in the GCL-inner nuclear layer (INL) in the sham group and showed the strongest fluorescence in the GCL layer and only weak fluorescence in the inner plexiform layer (IPL) and INL. After ONI, PirB expression in the retina of the ONI group was significantly higher than that in the sham group on the 1st day; the fluorescence signal was the strongest on the 7th day, and a strong fluorescence signal could be observed from GCL to outer nuclear layer (ONL), in which the fluorescence in GCL layer was the strongest and that in outer plexiform layer (OPL) layer was the weakest (Fig. 1A).

Western blotting results of retinal tissue showed that PirB expression in the ONI group 1, 3 and 7 days after ONI (0.49 ± 0.11, 0.38 ± 0.08, 0.43 ± 0.06, respectively) was higher than that in the blank (0.2 ± 0.06) and sham groups (0.25 ± 0.04, 0.18 ± 0.04, 0.24 ± 0.07, respectively), and the difference were statistically significant, all *p* < 0.05 (Fig. 1B).

### *PirB* knockdown by AAV PirB shRNA in the retina was successful

**Fig. 2:**
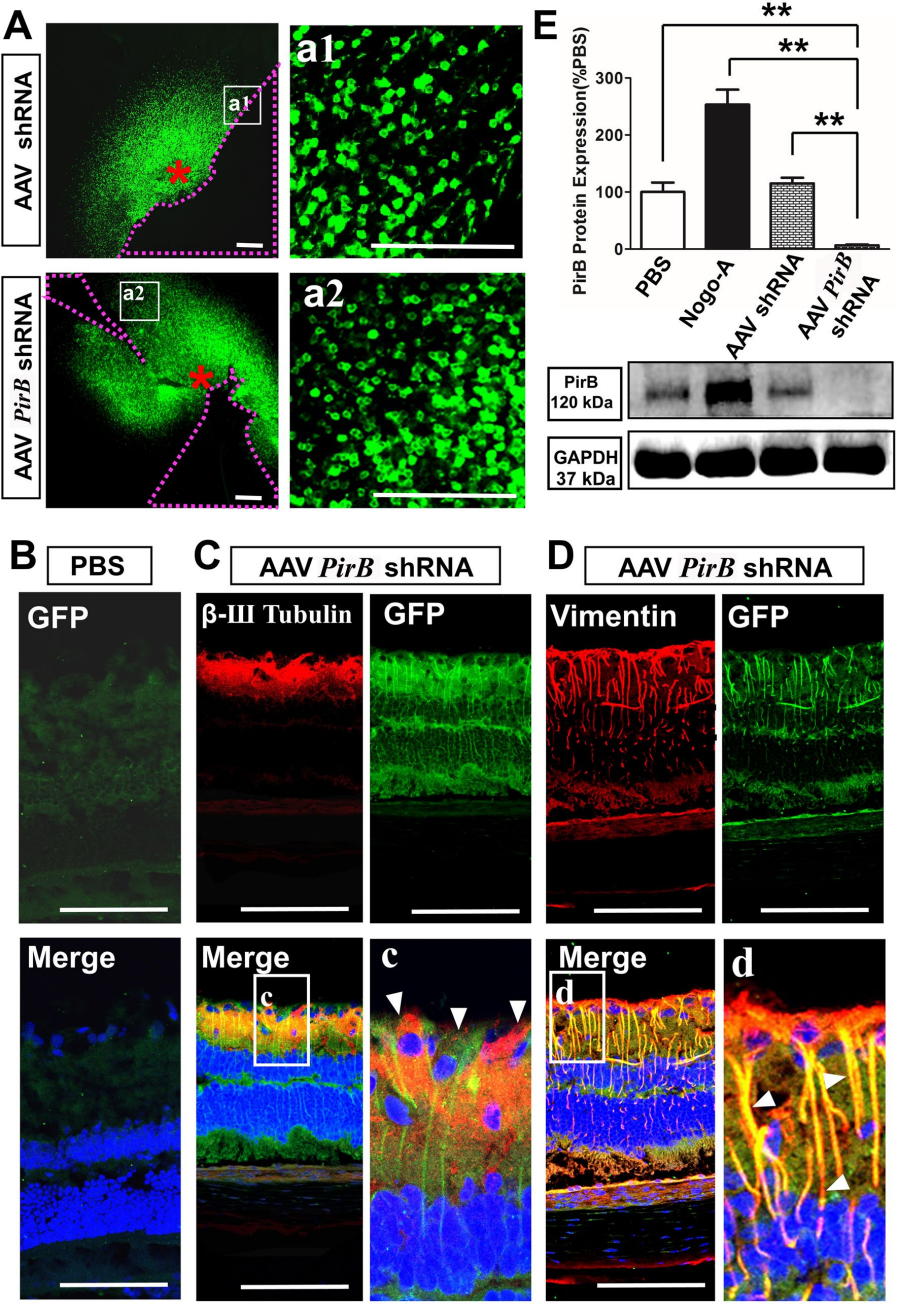
The effect of *PirB* knockdown in the retina by AAV *PirB* shRNA. **A** GFP (green, 488) expression in the retina transfected with AAV *PirB* shRNA or AAV shRNA for 21 days. red * represents the injection site, and the purple dotted line shows the area without the retinal tissue. Scale bars: 150 μm. **B** GFP staining in the PBS group. DAPI represents nuclear staining (blue fluorescence). Scale bar: 250 μm. **C** GFP (green, 488) and β-Ш Tubulin (red, 594) fluorescent staining in the AAV *PirB* shRNA group. DAPI represents nuclear staining (blue). Scale bar: 250 μm. Small white arrows in c show GFP and β-Ш Tubulin co-localization. **D** GFP (green, 488) and Vimentin (red, 594) fluorescent staining in the AAV *PirB* shRNA group. DAPI represents nuclear staining (blue). Scale bar: 250 μm. Small white arrows in d show GFP and Vimentin co-localization. **E** Bar charts showing PirB expression in the retina tissue. GAPDH was used as an internal reference. The ratios of gray values of target proteins to those of respective internal references are provided. The experiment was repeated three times (n = 4). Data represent the mean ± standard deviation. One-way ANOVA was performed, and Student’s *t*-test was used for group-pair comparisons used. **, *p* < 0.01

After 21 days of intravitreal injection of AAV shRNA (empty virus control) or AAV *PirB* shRNA, large numbers of cells scattered virus green fluorescent protein (GFP) in the retina of the two groups were observed, which radiated around the injection point (red *) and gradually weakened in all directions. The positive fluorescence was mainly present in the cytoplasm of the cells (Fig. 2A).

After 21 days of PBS or AAV *PirB* shRNA injection, the GFP signal of the virus was not detected in the retina of the PBS group (negative control) (Fig. 2B). In the AAV *PirB* shRNA group, GFP was expressed in RGCs, which co-localized with β-tubulin (specific for RGC) (Fig. 2C). GFP was also expressed in Müller cells in the same way and co-localized with vimentin (specific for Müller cells) (Fig. 2D).

Western blotting results of the retina tissue after 21 days of PBS, Nogo-A (positive control), AAV shRNA, or AAV *PirB* shRNA injection showed that PirB expression in the AAV *PirB* shRNA group (6.72 ± 1.44) was significantly lower than that in the PBS (100 ± 16.65, *p* = 0.007), Nogo-A (253.48 ± 25.93, *p* = 0.001), and AAV shRNA groups (115.13 ± 10.07, *p* = 0.009) (Fig. 2E). These results indicated that AAV *PirB* shRNA significantly knocked down *PirB* expression in the retina.

### *PirB* knockdown promotes the level of P-Stat3 and expression of CNTF in the retina after ONI

**Fig. 3:**
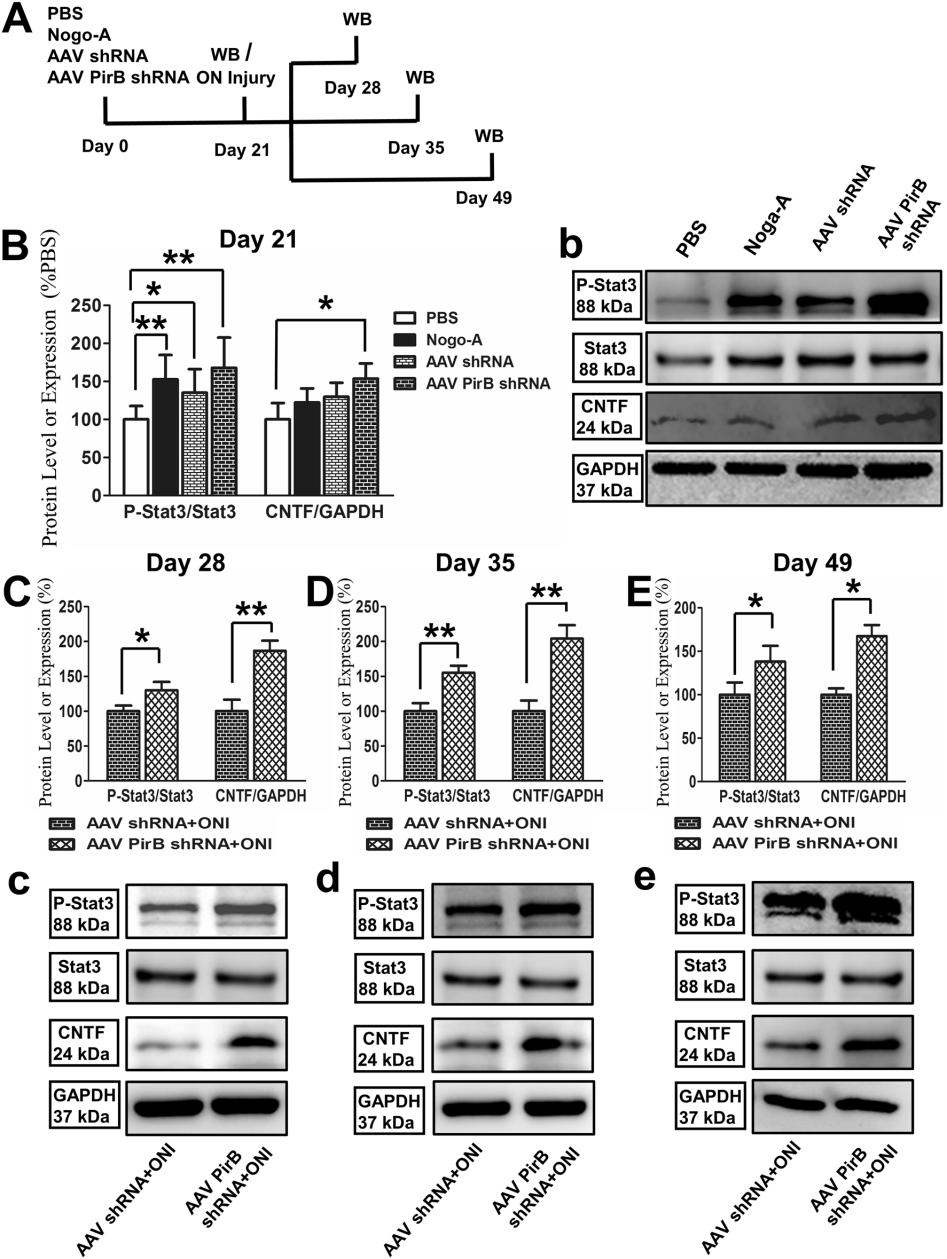
Expressions of P-Stat3 and CNTF in the retina of each group. **A** Time course of the treatment for each group of animals. WB, western blotting; ON, optical nerve. **B** P-Stat3 leve and CNTF expressions 21 days after intravitreal injection in four groups. **C** P-Stat3 level and CNTF expressions 28 days after intravitreal injection in AAV shRNA + ONI and AAV *PirB* shRNA + ONI groups, **D** P-Stat3 level and CNTF expressions 35 days after intravitreal injection in AAV shRNA + ONI and AAV *PirB* shRNA + ONI groups. **E** P-Stat3 level and CNTF expressions 49 days after intravitreal injection in AAV shRNA + ONI and AAV *PirB* shRNA + ONI groups. GAPDH and Stat3 were used as internal references. Original blots are shown in c, d, and e. The ratios of gray values of target proteins to those of respective internal references are provided. The experiment was repeated three times (n = 4). Data are presented as the mean ± standard deviation. One-way ANOVA was performed, and Student’s *t*-test was used for group-pair comparisons. *p < 0.05 and **p < 0.01

Rats were treated as previously described in the methods (Fig. 3A). Retinal tissues were extracted for western blotting 21 (before ONI), 28 (7 days after ONI), 35 (14 days after ONI), and 49 days (28 days after ONI) after intravitreal injection.

Western blotting results showed that 21 days after intravitreal injection, the level of P-Stat3 in Nogo-A (159.63 ± 25.39, *p* = 0.001), AAV shRNA (135.02 ± 29.22, *p* = 0.041), and AAV *PirB* shRNA (167.9 ± 35.85, *p* = 0.001) groups was significantly higher than that in the PBS group (100 ± 15.6). The expression of CNTF in the AAV *PirB* shRNA group (143.71 ± 19.86, *p* = 0.035) was significantly higher than that in the PBS group (100 ± 21.57) (Fig. 3B, b).

Precisely 28 days after intravitreal injection (7 days after ONI), level of P-Stat3 (130.76 ± 9.95, P = 0.047) and expressions of CNTF (186.78 ± 14.37, *p* = 0.001) in the AAV *PirB* shRNA + ONI group were significantly (*p* < 0.05, *p* < 0.01, respectively) higher than those in the AAV shRNA + ONI group (100 ± 8.58; 100 ± 16.51) (Fig. 3C, c).

Exactly 35 days after intravitreal injection (14 days after ONI), level of P-Stat3 (155.3 ± 9.43, *p* = 0.001) and expressions of CNTF (203.96 ± 19.57, P = 0.001) in the AAV *PirB* shRNA + ONI group were significantly (*p* < 0.01) higher than those in the AAV shRNA + ONI group (100 ± 13.3; 100 ± 15.24) (Fig. 3D, d).

Moreover, 49 days after intravitreal injection (28 days after ONI), level of P-Stat3 (138.22 ± 15.37, *p* = 0.048) and expressions of CNTF (167.43 ± 12.76, *p* = 0.027) in the AAV *PirB* shRNA + ONI group were significantly higher than those in the AAV shRNA + ONI group (100 ± 14.13; 100 ± 7.36) (Fig. 3E, e).

### *PirB* knockdown has no significant protective effect on RGC survival after ONI

**Fig. 4:**
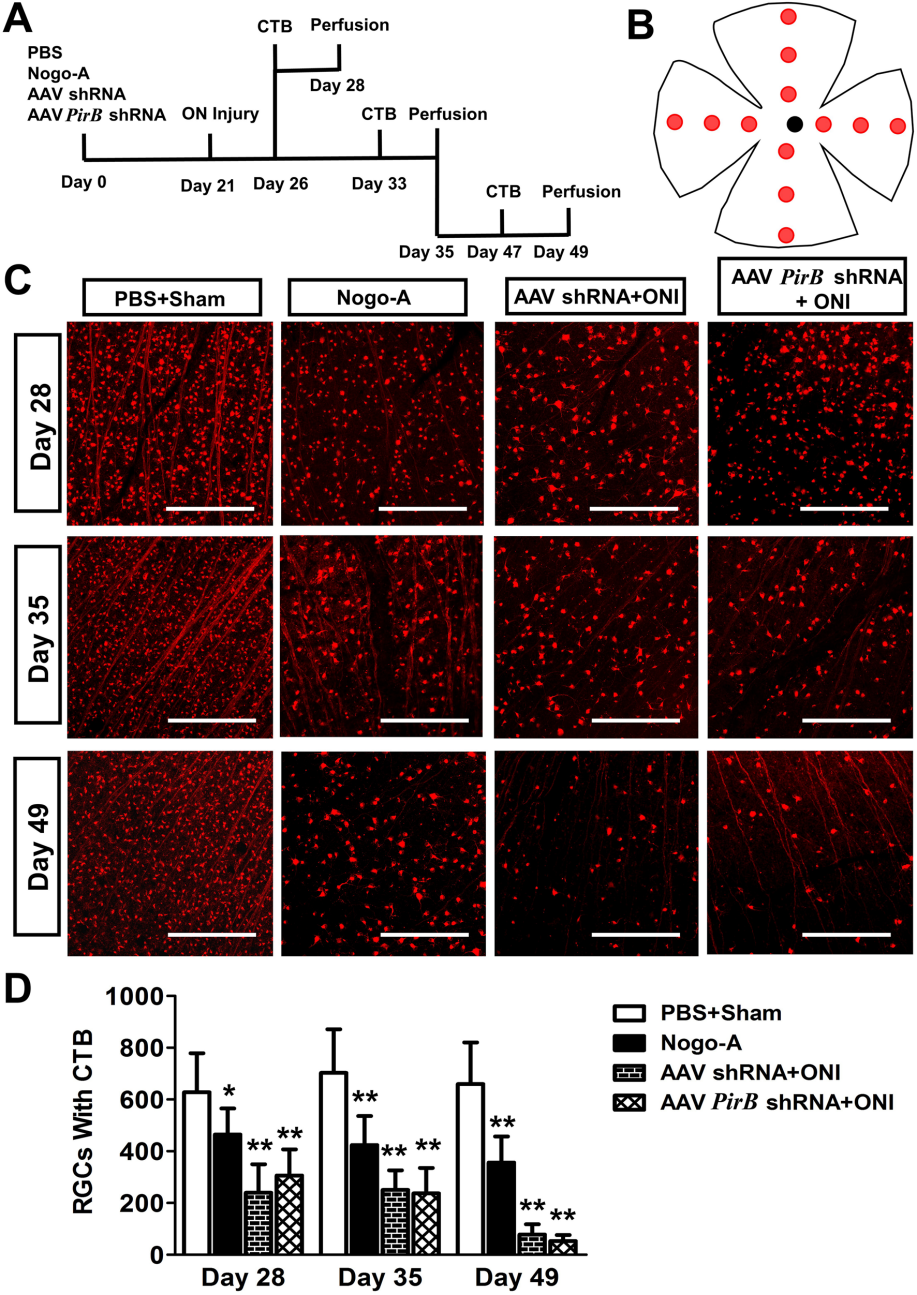
The number of CTB-positive labeled RGCs in the retina. **A** Time course of treatment for each group of animals. CTB, cholera toxin subunit B; ON, optic nerve. **B** Schematic diagram of the detection areas used for laser confocal microscopy. The red solid circle represents the selected imaging area, and the black solid circle represents the optic nipple. **C** Representative photomicrographs showing the CTB-positive RGCs (red, 594) in the retina for each group. **D** Bar charts showing the RGC count in the retina for each group. ONI: optic nerve injury. Scale bar: 250 μm, * compared with the PBS + sham group *p* ˂ 0.05, ** compared with the PBS + sham group *p* ˂ 0.01, n = 4. ANOVA followed by a post hoc Student’s t-test were used

Rats were treated as described in methods (Fig. 4A). The retinas were removed on the 28th, 35th and 49th day after injection. The images of 12 areas of each retinal patch were acquired using a laser confocal microscope, and the number of CTB-positive (red) RGCs in each image was counted (Fig. 4B).

The results of RGC counts showed that on the 28th day after injection (7 days after ONI), the cell counts in Nogo-A (464.42 ± 100.75, *p* = 0.045), AAV shRNA + ONI (219.93 ± 109.80, *p* = 0.004), and AAV *PirB* shRNA + ONI (275.07 ± 109.4, *p* = 0.009) groups decreased significantly compared with those in the PBS + sham group (628.28 ± 150.31).

Precisely 35 days after injection (14 days after ONI). The cell counts in Nogo-A (423.51 ± 112.74, *p* = 0.003), AAV shRNA + ONI (250.55 ± 75.86, *p* = 0.001), and AAV *PirB* shRNA + ONI (237.14 ± 98.62, *p* = 0.001) groups decreased significantly (all *p* < 0.01) compared those in the PBS + sham group (702.78 ± 168.37).

Moreover, 49 days after injection (28 days after ONI). The cell counts in Nogo-A (356.18 ± 100.75, *p* = 0.002), AAV shRNA + ONI (77.93 ± 39.64, *p* = 0.001), and AAV *PirB* shRNA + ONI (53.18 ± 23.43, *p* = 0.001) groups decreased significantly (all *p* < 0.01) compared with those in the PBS + sham group (652.22 ± 159.45).

There was no significant difference between the cell counts of AAV shRNA + ONI and AAV *PirB* shRNA + ONI groups ( *p* > 0.05), indicating that *PirB* knockdown had no significant protective effect on RGC survival (Fig. 4C, D).

### *PirB* knockdown in the retina promotes axonal regeneration after ONI

**Fig. 5:**
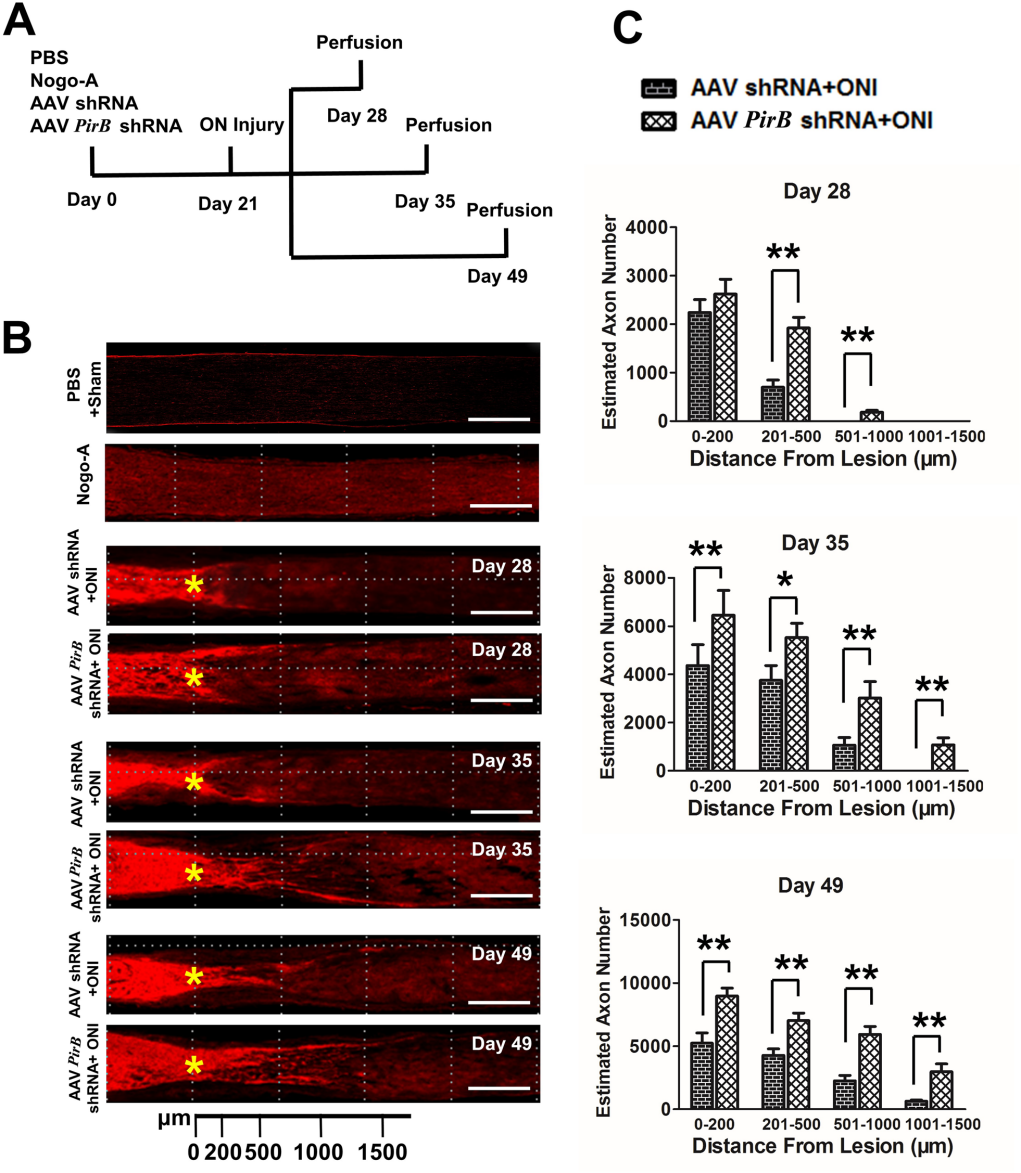
GAP43 expression representing the regenerated ON fibers in each group. **A** Time course of treatment for each group of animals. ON, optic nerve. **B** Representative photomicrographs showing the injury site and regenerating axons via GAP 43 (red, 594) immunofluorescence staining. Yellow * stands for ONI site. Scale bar: 500 μm. **C** Bar charts showing the regenerating axons on the right of the ONI site in each group. ONI: optic nerve injury, *p ˂ 0.05, **p ˂ 0.01, n = 4. The student’s t-test was used

Rats were treated as previously described in methods (Fig. 5A). Precisely 28, 35 and 49 days after injection, the ONs were removed for frozen section preparation and stained for growth-associated protein 43 (GAP43) (red, 594). The results of immunofluorescence of the ON showed that there was no obvious expression of GAP43 in the PBS + sham group (negative control); GAP43 expression in the Nogo-A group (positive control) was scattered along the whole longitudinal section of the ON; AAV shRNA + ONI and AAV *PirB* shRNA + ONI groups expressed GAP43, and the fluorescence intensity showed obvious two-stage differentiation at both ends of the injury site. The fluorescence signal on the left (eyeball end) of the injury site was strong and dense while that on the right (optic chiasm end) was significantly weakened and sparsely distributed. The farther the objective moved from the injury site, the weaker the fluorescence intensity became until it disappeared (Fig. 5B).

The number of regenerated nerve fibers (optic chiasm end) 28 days after the injection of the adenovirus (7 days after ONI) in the AAV *PirB* shRNA + ONI group was significantly (1920.86 ± 221.37, *p* = 0.001; 181.65 ± 41.26, *p* = 0.000) higher than that in the AAV shRNA + ONI group with a length of 201–500 μm (699.88 ± 147.32) and 501–1000 μm (0).

On the 35th day after adenovirus injection (14 days after ONI), the number of regenerated nerve fibers (optic chiasm end) with a length of 0–200 μm (6456.00 ± 1023.55, *p* = 0.001), 201–500 μm (5527.18 ± 596.32, *p* = 0.023), 501–1000 μm (3021.84 ± 682.19, *p* = 0.001), and 1001–1500 μm (1079.56 ± 290.12, *p* = 0.000) in the AAV *PirB* shRNA + ONI group was significantly (*p* < 0.05, *p* < 0.01) higher than those in the AAV shRNA + ONI group (4368.17 ± 868.31, 3756.44 ± 614.59, 1062.35 ± 318.44, 0, respectively).

On the 49th day after adenovirus injection (28 days after ONI), the number of regenerated nerve fibers (optic chiasm end) with a length of 0–200 μm (8971.42 ± 617.83, *p* = 0.001), 201–500 μm (7018.22 ± 597.31, *p* = 0.001), 501–1000 μm (5935.47 ± 618.57, *p* = 0.001), and 1001–1500 μm (2962.53 ± 627.84, *p* = 0.001) in the AAV *PirB* shRNA + ONI group was significantly (all *p* < 0.01) higher than those in the AAV shRNA + ONI group (5233.65 ± 806.5, 4269.58 ± 517.86, 2257.68 ± 423.72, 625.75 ± 108.54, respectively) (Fig. 5C).

### *PirB* knockdown in the retina improved the N1-P1 and P1-N2 amplitudes after ONI

**Fig. 6:**
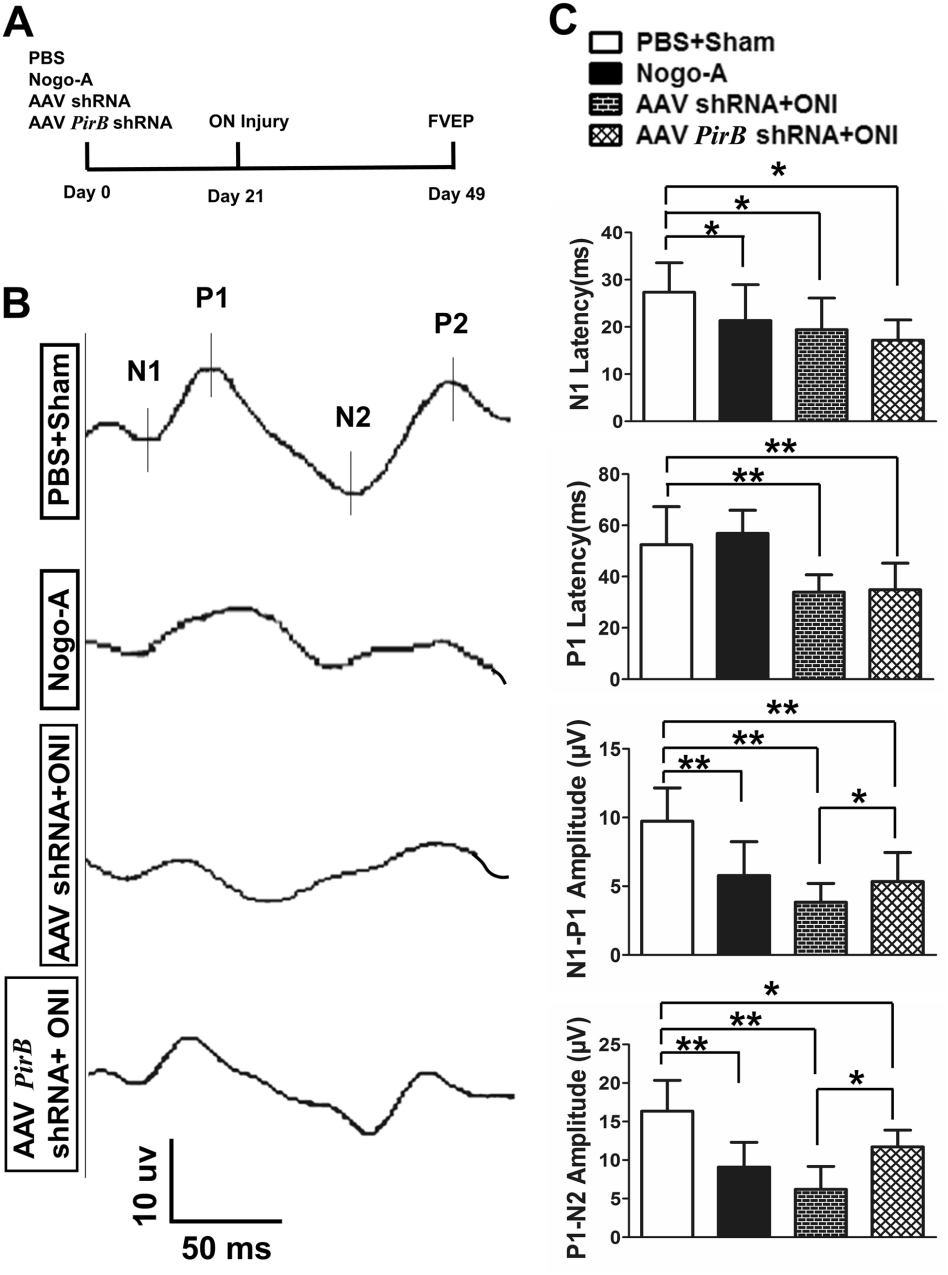
Visual function of rats detected via FVEP. **A** Time course of treatment for each group of animals. ON, optical nerve; FVEP, flash visual evoked potential. **B** Representative FVEP wavelength in each group. **C** Bar charts showing N1 and P1 latency, as well as N1- P1, P1-N2 amplitude in each group. ONI: optic nerve injury, *p ˂ 0.05, **p ˂ 0.01, n = 4, the one—way ANOVA and Student’s t-test

Rats were treated as previously described in the methods (Fig. 6A). Visual function was examined via FVEP 49 days after intravitreal injection (28 days after ONI). FVEP of Nogo-A, AAV shRNA + ONI, and AAV *PirB* shRNA + ONI groups was lower than that of the PBS + sham group (Fig. 6B).

The N1 latency of Nogo-A (21.36 ± 7.58, *p* = 0.042), AAV shRNA + ONI (19.43 ± 6.7, *p* = 0.037), and AAV *PirB* shRNA + ONI (17.19 ± 4.3, *p* = 0.022) groups was significantly (all *p* < 0.05) shorter than that of the PBS + sham group (27.36 ± 6.21) (Fig. 6C).

The P1 latency of AAV shRNA + ONI (33.87 ± 6.81, *p* = 0.002) and AAV *PirB* shRNA + ONI (34.81 ± 10.41, *p* = 0.001) groups was significantly (*p* < 0.01) shorter than that of the PBS + sham group (52.45 ± 14.84) (Fig. 6C).

The N1-P1 amplitudes of Nogo-A (5.775 ± 2.47, *p* = 0.002), AAV shRNA + ONI (3.825 ± 1.38, *p* = 0.001), and AAV *PirB* shRNA + ONI (5.35 ± 2.11, *p* = 0.001) groups were significantly (all *p* < 0.01) lower than those of the PBS + sham group (9.725 ± 2.43); the N1-P1 amplitudes of AAV shRNA + ONI group were significantly (*p* = 0.047) lower than those of AAV *PirB* shRNA group (Fig. 6C).

The P1-N2 amplitudes of Nogo-A (9.1 ± 3.21, *p* = 0.002), AAV shRNA + ONI (6.19 ± 2.98, *p* = 0.001), and AAV PirB shRNA + ONI (11.71 ± 2.17, *p* = 0.48) groups were significantly (*p* < 0.05 or *p* < 0.01) lower than those of the PBS + sham group (16.334 ± 4.02); the P1-N2 amplitudes of AAV shRNA + ONI group were significantly (*p* = 0.039) lower than those of AAV PirB shRNA group (Fig. 6C).

## Discussion

Previously we found that PirB is expressed in GCL, INL, and ONL of mouse retina in vivo, and in primary culture of RGCs and Müller cells of newborn SD rat retina in vitro [19]. In the present study, we found that PirB was expressed in the retina of adult rats, mainly in the GCL under normal conditions, and the expression of PirB gradually extended from GCL to ONL with the extension of injury time after ONI (Fig. 1A). The results showed that the distribution of PirB expression in rat and mice retinas was highly similar, and confirmed that PirB was one of the pathological molecules, which might play an important role in the pathogenesis of ONI.

We also wanted to reveal the pathological role of PirB in the retina after ONI, as well as the effect of inhibiting PirB expression on optic nerve repair and regeneration. In this study, AAV *PirB* shRNA was injected into the vitreous cavity for the successful localized *PirB* knock down. It is easier to operate than directly inject AAV into the ON or the tissue around the ON [20, 26], as surgery allows direct manipulation of the RGC cell body and maintains the *PirB* knockdown effect for a long time without causing additional damage to the ON. In general, the level of the corresponding receptor of this ligand will decrease with the increase of the ligand level. Therefore, Nogo-A was injected into the vitreous cavity as a positive control group that reduced PirB expression. However, we detected that the expression of PirB protein in the retina was significantly increased after intravitreal injection of exogenous Nogo-A (Fig. 2E). We speculate that there may be two likely reasons for this abnormal phenomenon: (1). PirB is not the only receptor of Nogo-A, such as NgR and S1P2R are also the receptors of Nogo-A [27, 28]. Their binding capacity with Nogo-A may be stronger than that of PirB, and competitively combining with exogenous Nogo-A to reduce the consumption of PirB. (2). The effect of exogenous Nogo-A and PirB may not last until 21 days after intravitreal injection. Nogo-A is one of the myelin-associated inhibitors, and it harmful to the neurons and axons of the retina [19, 29]. It is possible that the intravitreal injection of Nogo-A causes the activation of the internal signaling pathway in retinal cells to promote the expression of PirB protein.

We found that there was no significant difference in the RGC number between AAV *PirB* shRNA + ONI and AAV shRNA + ONI groups 7, 14, and 28 days after ONI (Fig. 4C, D). The number of new axons labeled with GAP43 in the AAV *PirB* shRNA + ONI group was more than that in the AAV shRNA + ONI group 7, 14 and 28 days after ONI. This suggested that *PirB* knockdown had no significant protective effect on the survival of RGCs, but promoted the regeneration of ON axons. It also suggested that the main pathological role of retinal PirB after ONI was to inhibit the regeneration of RGCs axons rather than promote the death of RGCs. It is worth mentioning that we found that the number of new axons at both ends of the ONI site had apparent two-stage differentiation. This phenomenon has also been reported in other studies, which indicates that there are one or more factors at the site of ONI that hinder the regeneration of nerve axons. This suggested that knock down PirB expression while targeting interventions for these factors might play a more significant role in promoting the regeneration of ON [13, 26].

The purpose of tissue remodeling is to achieve functional recovery and reconstruction. Similarly, the purpose of promoting RGC axon regeneration is also to protect and restore visual function. Visual acuity, visual field, color vision, stereoacuity, and visual evoked potential are important indicators of visual function^.^ [30–33] When the research objects are animals, it is difficult to cooperate the detection of vision, visual field, color vision, and stereoacuity. Instead, the FVEP under general anesthesia is one of the most advantageous visual function detection methods [34, 35]. People often evaluate the visual function of animals by detecting the latencies of N1 and P1 waves of FVEP, as well as N1-P1 and P1-N2 amplitudes [36, 37]. Here, we found that *PirB* knockdown did not significantly improve the latencies of N1 and P1 waves in FVEP, but significantly improved N1-P1 and P1-N2 amplitudes. Previous studies have found that latency of FVEP reflects the conduction of the ON, while amplitude of FVEP reflects the functional state of the axon [38, 39]. Therefore, we speculated that *PirB* knockdown might have a positive effect on axonal regeneration, but its effect on improving visual function was not ideal.

In our previous experiments, we explored the mechanism of how PirB promotes axonal regeneration. The results showed that PirB of Müller cells promotes axonal regeneration of co-cultured RGCs through JAK/Stat3 signaling pathway to regulate the expression of CNTF[19] In the present study, we found that *PirB* knockdown promoted the expression of P-Stat3 and CNTF in retina. In other words, *PirB* knockdown had a significant effect on the JAK/Stat3 pathway, promoting ON regeneration, which was consistent with our previous results. These results suggested that PirB might play an important role in the regulation of the JAK/Stat3 pathway after ONI. However, there are several cell types in the retina and our current research results do not clarify that axonal regeneration is achieved by PirB expressed in Müller cells, unless this deficiency can be overcome by achieving *PirB* knockout in retinal Müller cells, which will be our next research direction.

## Conclusions

In conclusion, the increase of retinal PirB expression after nerve injury is one of the important factors for inhibiting ON axon regeneration. Knockdown of retinal *PirB* significantly promotes ON axon regeneration, but its effect on improving visual function is not ideal. Combined intervention of PirB and other factors hindering ON regeneration may be better to promote ON regeneration and functional repair. This finding is expected to provide an experimental basis for PirB as a therapeutic target of promoting ON regeneration.

## Data Availability

The datasets used and/or analyzed during the current study are available from the corresponding author on reasonable request.

## Abbreviations

AAV: Adeno-associated virus
DAPI: 4′,6-Diamidino-2-phenylindole
CNTF: Ciliary neurotrophic factor
CNS: Central nervous system
CTB: Cholera toxin subunit B
FVEP: Flash visual evoked potential
GAP43: Growth associated protein-43
MHC I: Major histocompatibility complex class I
MAG: Myelin-associated glycoprotein
MAI: Myelin-associated inhibitor
NgR: Nogo receptor
OMgp: Oligodendrocyte-myelin glycoprotein
ONI: Optic nerve injury
PirB: Paired immunoglobulin-like receptor B
PBS: Phosphate buffered saline
RGC: Retinal ganglion cells
SD: Sprague–Dawley
